# Evaluating the relationships among stress, resilience and psychological well-being among young adults: a structural equation modelling approach

**DOI:** 10.1186/s12912-021-00645-9

**Published:** 2021-07-05

**Authors:** Piyanee Klainin-Yobas, Nopporn Vongsirimas, Debbie Q. Ramirez, Jenneth Sarmiento, Zenaida Fernandez

**Affiliations:** 1 Alice Lee Centre for Nursing Studies, National University of Singapore, Level 2, Clinical Research Centre, Block MD 11, 10 Medical Drive, Singapore, 117597 Singapore; 2 Department of Mental Health and Psychiatric Nursing, Faculty of Nursing, Mahidol University, 2 Pran-nok Road, Bangkoknoi, Bangkok, 10700 Thailand; 3 College of Nursing, Angeles University Foundation, MacArthur Highway, 2009 Angeles City, Central Luzon Philippines

**Keywords:** Autonomy and growth, Negative triad, Psychological well-being, Resilience, Stress, University students

## Abstract

**Background:**

Resilience reflects individuals’ ability to bounce back quickly in the face of stressful situations. Resilience is positively correlated with psychological well-being (PWB) and negatively related to poor mental health. However, there is limited longitudinal research to confirm the causal relationships between resilience and PWB. This study aimed to examine the relationships among stress, resilience, and PWB among youths in the Philippines across two samples. A descriptive comparative study was conducted and two repeated cross-sectional samples were recruited. Eligible participants were undergraduate students from a university in the Philippines regardless of sociological backgrounds. Data were collected via anonymous online questionnaires; and analyzed by using descriptive statistics and structural equation modelling (SEM).

**Results:**

A total of 630 were recruited (Sample 1 = 221 and Sample 2 = 409). Most of whom were female, Filipino, Christian and students from Nursing School. Results from SEM indicated that the hypothesized two-group models had an adequate fit with sample data. Furthermore, perceived control and resilience were significant predictors of the autonomy & growth factor of PWB. Perceived stress and resilience significantly predicted the negative triad factor of PWB. These findings were comparable across the two samples providing strong evidence to support causal relationships among the study variables.

**Conclusion:**

There is a need to offer stress management interventions and resilience-based programs to enhance PWB. Additional research should be conducted to test the efficacy of the interventions.

## Background

Resilience is a crucial concept to help individuals achieve psychological well-being (PWB). Thus far, there is no consensus about the definition of resilience. However, a literature review summarized that the definition of resilience has three orientations: personality trait, outcome, and process [1]. As a personality trait, resilience is postulated to enhance individuals’ ability to cope with adversity and to achieve positive adaptation [2]. Resilience is also a behavioral outcome that can help people deal with adversity [3]. As a process, resilience is viewed as an interactive process that contribute to positive health outcomes, even in the face of stressors and environmental challenges [4]. Different sources are perceived to contribute to the development of resilience: personal, biological, and environmental-systematic factors [5]. Personal factors include personality trait, cognitive appraisal, internal locus of control, mastery, self-efficacy, self-esteem, and optimism [5]. Biological factors are a brain structure and neurobiological system [5]. Environment-systematic factors entail social support, relationships with others and community services [5]. A systematic review of 60 studies showed that trait resilience had a negative correlation with negative mental health, with an average correlation coefficient (r) of − 0.36 [1]. Furthermore, resilience had a positive correlation with indicators of positive mental health and PWB such as positive affect and satisfaction with life (*r* = 0.50) [1].

World Health Organization (WHO) emphasizes that health is the state of welling, including physical, social and mental (psychological) well-being [6]. The state of psychological well-being (PWB) is of importance as it is linked to the prevention and recovery of physical conditions [7]. PWB refers to the absence of mental health problems and the presence of self-acceptance (a positive self-evaluation), personal growth, purpose in life (belief that a person has a purposeful and meaningful life), positive relations with others, environmental mastery (an ability to manage life and environment), and autonomy (a sense of determination) [8, 9]. A study conducted on undergraduate university students in the Philippines revealed that PWB had two major dimensions: positive PWB and negative PWB and these two dimensions resulted from a series of factor analyses [10]. Among undergraduate students, mindfulness; self-efficacy; and social support from family, friends, and significant others are significant predictors of positive PWB and negative PWB [10].

University students are the future of all nations and healthy students would contribute to future powerful workforces. However, they are perceived to experience stressors that affect their health and PWB [11]. Stress refers to an array of cognitive, emotional, physiological, and behavioral reactions to perceived undesirable situations [12]. Stress takes place when a person appraises a situation as a threat that exceeds his/her available coping resources [13]. This may trigger negative emotions such as anger, anxiety, fright, guilt, shame, envy, jealousy, disgust and sadness [14]. University students may face various stressful circumstances relating to their academic (such as examinations and assignments), family (such as family relationships and financial problems), social (such as relationships with friends), and developmental matters (such as biological changes and transition from childhood to adulthood) [11, 15, 16]. Studies showed that stress may lead to poorer PWB [17], mental distress [18] (Tesfaye, 2009) and other mental disorders such as eating disorder [19]. There is a need to examine how resilience can help university students survive stressful situations and achieve PWB.

Several studies examined the relationships among stress, resilience, and PWB among undergraduate students. A systematic review (involving 12 studies) suggested that PWB had significant relationships with stress and resilience among nursing students [20]. A non-experimental study in China involving 2925 medical students revealed that life satisfaction, one of the measures of PWB, was negatively correlated with stress and positively correlated with resilience [21]. Furthermore, resilience mediated the effects of stress on life satisfaction [21]. Another study in Iran found that perceived negative stress, perceived positive stress, and resilience were significant predictors of life satisfaction among two groups of students: success and failure ones [22]. Furthermore, all predictors explained 31 and 49% of variance on success and failure students respectively [22].

### The current study

This study aimed to examine and compare the predicting effects of stress and resilience on PWB across two repeated cross-sectional samples. We hypothesized that: a) stress would have a significant negative effect on PWB among university students in the Philippines, b) resilience would have a positive effect on PWB, and c) the magnitude of the effects would be comparable across the two samples.

**Fig. 1:**
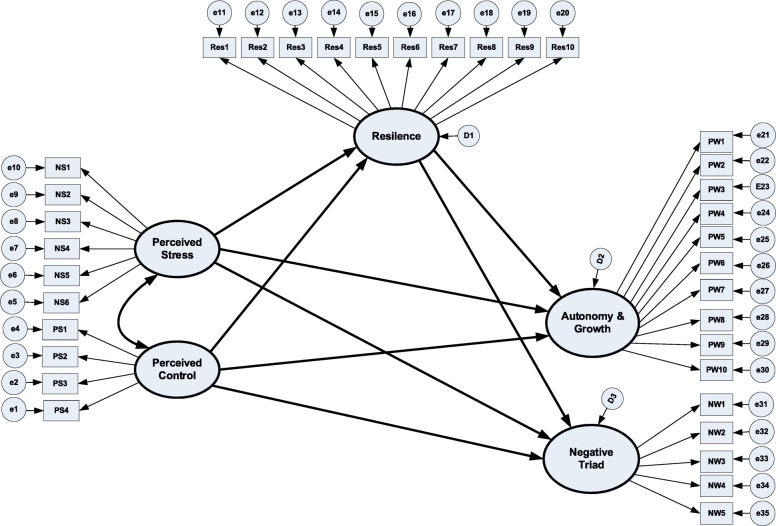
Hypothesized model for Sample 1 and Sample 2. **a** Ellipses represent study variables, Boxes represent questionnaire items, Circles represent error variances. **b** Arrows linking ellipses are regression paths, Arrows linking ellipses and boxes are factor loadings, Double-arrowed line is covariance, **c** Parameters to be estimated = 82 (8 regression paths, 35 factors loadings, 38 error variances, and 1 covariance); Degree of freedom = 549

The hypothesized model for Samples 1 and 2 is displayed in Fig. 1. In this Figure, ellipses represent study variables, boxes represent questionnaire items, and circles represent error variances. Furthermore, arrows linking ellipses are regression paths, arrows linking ellipses and boxes are factor loadings, and double-arrowed lines represent correlation between study variables. During preliminary analyses, we did a series of exploratory and confirmatory factor analyses to examine factor structures of each measurement. Modification index (a feature in AMOS software) was used to determine if paths and/or measurement items should be included in the model. Results indicated that the measurement of resilience had one factor whereas stress had two factors: Perceived Stress and Perceived Control. PWB had two factors including Autonomy & Growth and Negative Triad. The factor structures of study variables are also displayed in Fig. 1.

Note that almost all existing studies used cross-sectional research design whereby the causal relationships among study variables cannot be confirmed. Therefore, this study minimizes such methodological limitation by collecting data twice to provide more solid evidence to support the relationships among study variables. Furthermore, we used structural equation modeling (SEM) to analyze data and it allowed a simultaneous analysis of measurement and structural models taken measurement errors into considerations. As such, results are perceived to be less bias. Moreover, we explored the factorial structure of each variable before testing the relationships; and, thus results would be more accurately estimated. Additionally, findings from this study adds knowledge and highlights the role of resilience in enhancing PWB. Hence, there are implications to clinical practice. Psychosocial interventions that strengthening participants’ resilience can be developed to minimize perceived stress and enhance two components of PWB (Autonomy & Growth and Negative Triad).

### Theoretical framework

This study is guided by the Psychological Well-being Promotion Model [23]. Within this model, there are four main constructs: stress, resource protection factors (RPFs), PWB, and prevention intervention. Stress is postulated to negatively affect PWB and RPFs protect individuals from the adverse effects of stress. RPFs are categorized as internal factors (such as resilience) and external factors (such as social support). In this study, we examined the relationships among resilience, stress and PWB among university students.

## Methods

### Study design

This research is part of a larger research examining the relationships among mindfulness, self-efficacy, social support and PWB on one sample [10]. In the current research, we used a comparative descriptive research design to test the hypothesized model linking stress, resilience, and PWB across two repeated cross-sectional samples (Fig. 1). This research design is appropriate as it allows exploring relationships among study variables and comparing findings across multiple samples in natural settings [24].

### Participants

Target participants were undergraduate students who were enrolled at a private university in the Philippines, which offered both graduate and undergraduate programs. For the latter, there were many schools/colleges, encompassing medicine, law, allied medical professions, art & sciences, business & accountancy, computer studies, criminal justice education, education, engineering & architecture, nursing and integrated schools. A convenience sampling was utilized to recruit potential participants and all undergraduate students were eligible for this study. Those who had a diagnosis of chronic medical conditions and/or mental disorders requiring hospitalization were excluded. Such health problems might be confounding factors as they might add stressful circumstances to participants under the study.

To determine an adequate sample size for this study, power analysis for structural equation modelling (SEM) [25] was utilized. As such, the hypothesized model (Fig. 1) was used to calculate the degree of freedom (df), a major component of power analysis. In doing so, the following formula was used: df = data point (D) – Unknown parameter (U) [26]. D was calculated by using the formula: D = p(p + 1)/2 where “p” is the number of observed variables or questionnaire items. As shown in Fig. 1, the number of questionnaire items is 35 and thus the resulting D would be 630 [(35 + 36)/2]. Furthermore, U was derived by adding all unknown parameters shown in Fig. 1 (comprising eight regression paths, 35 factor loadings, one correlation path and 38 error variances) and the resulting U would be 82. Therefore, the resulting *df* would be 548 (630–82).

Finally, four known parameters are required for the power analysis for SEM [25]. The parameters include: a) the desired power of 80%, b) statistical significance at α = 0.05, c) root mean square error of approximation (RMSEA) = 0.05 and 0.08 for the close fit and d) known value of df, which was 548 for the hypothesized model. Taken all the parameter together, the minimal sample size for the hypothesized model would be 132 for each sample [25].

### Data collection procedure

The entire study was carried out following ethical issues in accordance with the Declaration of Helsinki. It was commenced after receiving approval and ethical clearance from the Institutional Review Board (IRB) of Angelis University Foundation. All procedures were conducted according to the IRB guideline and regulations. Informed consent was obtained from all participants involved in this study. Data collection were between Year 2013 to 2015.

Afterward, we seek permission from Deans of schools/colleges to recruit potential participants at their respective schools/college and commenced data collection. Next, we sent an e-mail invitation to all undergraduate students, stating the purposes of the study and seek their participation. The Participant Information Sheet (PIS) was also attached with the e-mail. Interested participants were asked to complete online self-reported questionnaires, which took about 20–30 min (Sample 1). The issue of anonymity and voluntary participation were emphasized. Two follow-up e-mails were sent to all participants (regardless of their response) 1 week and 2 weeks respectively after the first mailout. A reminder message was provided to non-responders. The data collection repeated on undergraduate students 1 year later (Sample 2). The same documents including PIS were used.

### Variables and measurements

This research used online self-reported questionnaires to collect data. Each questionnaire contained the following measurements.

Stress was assessed by the 10-item Perceived Stress Scale (PSS) [27], capturing respondents’ thoughts and feelings during the last month. Items are designed to tap how unpredictable, uncontrollable, and overloaded respondents perceive their lives. The PSS has five response categories varying from 0 (never) to 4 (very often). For the current sample, the factor analyses showed that the PSS has two main factors: perceived stress and positive control and this evidence supported the construct validity of the scale. The perceived stress factor contained six items and the total score ranged from 6 to 24, with the highest score reflecting the highest level of stress. An example of this factor is “How often have you been upset because of something that happened unexpectedly?” The perceived control factor had four items and the summative score ranged from 4 to 16, with the highest score signifying the lowest level of control. An example of this factor is “How often have you felt confident about your ability to handle your personal problems?” Cronbach’s alphas of the PSS on American university students were in the range of 0.84–0.86 [27]. For the current sample, Cronbach’s alpha was 0.83 and 0.73 for the perceived stress and perceived control factor respectively.

Resilience was measured with the 10-item Connor–Davidson Resilience Scale (CD-RISC) [2, 28]. Respondents are asked to rate on the 5-point scale varying from 0 (not true at all) to 4 (true all the time). Total scores range from 0 to 40, with the highest scores indicating the highest levels of resilience. Examples of the items are “I am able to adapt when changes occur” and “I am able to handle unpleasant or painful feelings like sadness, fear, and anger.” Construct validity of the scale was supported by results from factor analyses on university students in Spain (*n* = 770), which suggested that the CD-RISC has one factor [29]. Similarly, the one-factor structure was also found in the current sample. Cronbach’s alpha of the scale was 0.95 on American undergraduate students [28], suggesting excellent reliability. For the current sample, Cronbach’s alpha was 0.89.

Psychological well-being was measured with the 18-item Psychological well-being Scale (PWBS) [9] comprising six subscales: autonomy, environmental mastery, purpose in life, personal growth, positive relations with others, and self-acceptance. Students responded on one of six-point categories ranging from (1) strongly disagree to (6) strongly agree. For the current sample, the factor analyses revealed that the PWBS had two main factors: autonomy & growth and negative triad. The autonomy & growth factor comprised 10 items and scores were in the range of 10–60, with the highest score representing the highest level of autonomy & growth. An example of this factor is “In general, I feel I am in charge of the situation in which I live.” The negative triad contained five items and the summative scores ranged from 5 to 30 with the highest score reflecting the lowest level of negative triad. An example of this factor is “Maintaining close relationships has been difficult and frustrating for me.” Note that three items were excluded from the analyses given that they did not load strongly on any of the factor mentioned above. The three items were “Did not experience warm and trusting relationships” “Influenced by people with strong opinions” and “Lived life one day at a time.” For the current sample, Cronbach’s alpha of the autonomy & growth and negative triad factor was 0.77 and 0.73 respectively.

### Data analyses

Data collected via the online questionnaires were electronically transformed to SPSS version 22. Afterward, we performed univariate analyses to describe characteristics of study participants and study variables. Subsequently, we tested the study hypotheses that stress and resilience would have significant predicting effects on PWB and the magnitude of the predicting effects would be equivalent across the Samples 1 and 2. As such, we submitted the multi-group hypothesized model (Fig. 1) to IBM AMOS version 23 and simultaneously ran the following models: a) the unconstrained (baseline) model and b) equality constraint model. For the unconstrained model, all statistical parameters (such as regression coefficient and factor loadings) were freely estimated on each Sample 1 and Sample 2 without any equality constraint. For the equality constrained model, we imposed that the parameters (such as regression coefficient and factor loadings) were equivalent across groups. The unconstrained model would serve as the baseline reference to compare with other subsequent models.

We determined model fits through the following parameters: a) confirmatory fit index (CFI), Incremental Fit Index (IFT), Tucker-Luwis Index (TLI) > 0.90 as acceptable fit and > 0.95 as well-fit, and b) root mean square of error of approximation (RMSEA) < 0.05 as well-fit and < 0.08 as reasonable fit [26]. We used a difference in chi-square statistics (Δχ^2^) and difference in comparative fit index (ΔCFI) to determine if statistical parameters were equivalent across the Samples 1 and 2 [26]. Specifically, a significant Δχ^2^ at the probability of less than 0.05 would indicate that the equality constraint model was significantly different from the baseline model. This served as evidence to determine that the hypothesized models were completely non-equivalent across samples (i.e., statistical parameters of the Samples 1 and 2 were not equivalent) [26]. Additionally, ΔCFI that less than the value of 0.01 would indicate that statistical parameters were not equivalent across samples [26].

## Results

### Description of study participants

There were 221 and 409 students completed the online questionnaires for the Sample 1 and Sample 2 respectively, making up a total sample size of 630. This sample size was sufficient according to the power analysis. Descriptions of both samples are illustrated in Table 1. For the Sample 1, participants’ age ranged from 16 to 48 (Mean = 19.56, SD = 3.03). Most participants were female (75.10%, *n* = 166) and Filipino (89.10%, *n* = 197). For the Sample 2, participants’ age ranged from 16 to 48 (Mean = 19.56, SD = 2.68). Most participants were female (80.90%, *n* = 244) and Filipino (70.20%, *n* = 287).

**Table 1:** Descriptions of study participants

Variables	Sample 1 (*n* = 221)		Sample 2 (*n* = 409)	
	Frequency	Percentage (%)	Frequency	Percentage (%)
Gender				
Male	55	24.90	78	19.1
Female	166	75.10	331	80.9
Race				
American	9	4.10	14	3.4
Filipino	197	89.10	287	70.2
Filipino-American	2	0.90	8	2.0
Pacific Islander	1	0.50	4	1.0
Timorese	1	0.50	1	.2
Missing	11	5.00	94	23.0
School				
Allied Health	11	5.00	23	5.60
Art & Science	21	9.50	39	9.50
Business & Accountancy	17	7.70	37	9.00
Nursing & Medicine	172	77.80	310	75.80

### Descriptions of study variables

Descriptions of study variables are illustrated in Table 2. Data for the total sample, Sample 1, and Sample 2 are also reported. There were no violations of a normality assumption for all variables.

**Table 2:** Demographic information of study variables

Study Variables	Total Sample (*n* = 630)			Sample 1 (*n* = 221)			Sample 2 (*n* = 409)		
	Mean	SD^a^	n^a^	Mean	SD^a^	n^a^	Mean	SD^a^	n^a^
Perceived stress^b^	20.07	4.04	630	20.15	4.20	221	20.02	3.95	409
Perceived control^b^	10.04	2.32	630	10.11	2.40	221	10.01	2.28	409
Resilience	38.87	6.16	630	39.06	6.59	221	38.77	5.92	409
Autonomy & growth^c^	47.61	5.82	630	47.74	5.82	221	47.54	5.82	409
Negative triad^c^	21.83	4.56	630	21.77	4.82	221	21.86	4.42	409

a) SD = Standard deviation, *n* = sample size

b) Stress had two factors: Perceived stress and Perceived control

c) Psychological well-being (PWB) had two factors: Autonomy & growth and Negative triad

### Predictors of PWB across the samples 1 and 2

**Fig. 2:**
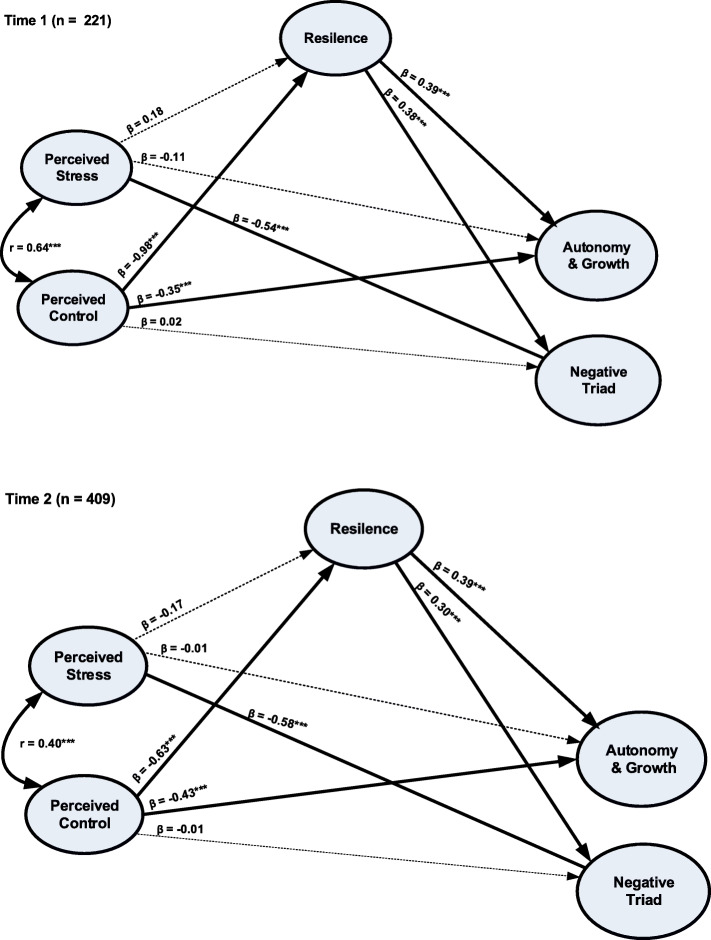
Predictors of psychological well-being for Sample 1 (Time 1) and Sample 2 (Time 2). **a** chi-square = 1499.93, degree of freedom = 1032, *p* = 0.000. **b** Incremental Fit Index = 0.939, Tucker Lewis Index = 0.928, Comparative Fit Index = 0.938. **c** Root mean square error of estimation (RMSEA) = 0.027, 95% CI RMSEA = 0.024–0.030. **d** ** = Significance level at α = 0.01, *** = Significance level at α = 0.001

The hypothesized models for the Samples 1 and 2 (the multiple-group model) were submitted to IBM AMOS version 23 and results are illustrated in Table 3 and Fig. 2. According to Table 2, the unconstrained model had χ^2^ = 1499.93 with 1032 degree of freedom (CFI = 0.938, RMSEA = 0.027). The equality constraint model had χ^2^ = 1576.97 with 1146 degree of freedom (CFI = 0.943, RMSEA = 0.024). Furthermore, Δχ^2^ between the two models was 77.04 with Δdf = 114 and this χ^2^ difference was statistically different at a probability of less than 0.05. This result indicated that statistical parameters (such as regression coefficient and factor loadings) were not equivalent across the two samples. Additionally, the ΔCFI value of 0.005 (< 0.01) further suggested that statistical parameters did not operate equally across the samples.

**Table 3:** Fit indices of structural equation models (SEM)

Model	Chi-square	Degree of freedom (df)	*p*-value	IFI^a^	TLI^a^	CFI^a^	RMSEA^a^	95% Confidence Interval of RMSEA^a^
Unconstrained model^b^	1499.93	1032	0.000	0.939	0.928	0.938	0.027	0.024–0.030
Constrained model (factor loadings and regression weights)^b^	1576.97	1146	0.000	0.943	0.941	0.943	0.024	0.021–0.027

a) *IFI* Incremental Fit Index, *TLI* Tucker-Lewis Index, *CFI* Comparative Fit Index, *RMSEA* Root Mean Square Error of Approximation

b) Difference in chi-square (Δχ^2^) between the unconstrained and constrained models = 77.04, Δdf = 114, *p* < 0.001, ΔIFI = 0.002, ΔTLI = 0.013, and ΔCFI = 0.005

Accordingly, findings from the unconstrained models were further used to test the study hypotheses. Specifically, predictors of psychological well-being are shown in Fig. 2. For the sample 1, perceived control (β = − 0.35, *p* < 0.001) and resilience (β = 0.39, *p* < 0.001) were significant predictors of the autonomy & growth factor of PWB. The magnitudes of the relationships were comparable across the two predictors. Perceived stress (β = − 0.54, *p* < 0.001) and resilience (β = 0.38, *p* < 0.001) were significant predictors of the negative triad factor of PWB. However, perceived stress had a stronger effect than resilience. Furthermore, perceived control (β = − 0.98, *p* < 0.001) was a significant predictor of resilience.

Predictors of PWB are shown in Fig. 2. For the sample 2, perceived control (β = − 0.43, *p* < 0.001) and resilience (β = 0.39, *p* < 0.001) were significant predictors of the autonomy & growth factor. Note that perceived control had a slightly stronger effect than resilience. Perceived stress (β = − 0.63, *p* < 0.001) and resilience (β = 0.30, *p* < 0.001) were significant predictors of the negative triad factor. Similar to sample 1, perceived stress had a stronger effect than resilience. Furthermore, perceived control (β = − 0.63, *p* < 0.001) was a significant predictor of resilience.

## Discussion

This study aimed to examine and compare the predicting effects of stress and resilience on PWB among undergraduate students across the two samples. Results from both samples suggested that students who had higher perceived control and greater resilience reported higher levels of autonomy and growth. Students who had lower perceived stress and greater resilience experienced lower levels of negative triad. Furthermore, the magnitudes of the predicting effects of perceived stress, perceived control, and resilience on PWB were not equivalent across the two samples but the differences were small. However, a marked difference was observed in the magnitude of the effect of perceived control on resilience, in which the Sample 1 had a greater predicting effect than that of the Sample 2. The difference might result from different gender compositions across the samples. Specifically, female youths accounted for 75.1 and 59.7% for the Samples 1 and 2 respectively. More male youths in the Sample 2 might add higher scores on perceived control and thus contributing to the stronger perceived control-resilience relationship. Future research may explore gender difference in the relationships among stress, resilience and PWB across time.

In this study, the measurement of stress (the perceived stress scale) contained two factors: perceived stress and perceived control. As expected, both factors were significant predictors of PWB among undergraduate students across the samples. Similarly, a study in China revealed that college stress (academic hassle, personal hassle, negative life event, and overall stress) were negatively related to PWB and positively associated with psychological distress among undergraduate students [17]. Such findings are not surprising given that stressful situations can trigger the body reactions, including cognitive, emotional, physiological, and behavioral ones [12]. It is well-established that stress activates a sympathetic nervous system (flight-or-fright reaction) and links to various health conditions such as coronary heart disease, atherosclerosis, hypertension, migraine headache, cancer, allergy, and peptic ulcers [12]. According to Lazarus (1993), stress may contribute to various negative emotions such as anger, anxiety, fright, guilt, shame, envy, jealousy, disgust, and sadness [14].

Our findings showed that resilience was associated with higher autonomy and growth and lower negative triad across the two repeated cross-sectional samples, approximately 1 year apart. This solid evidence helped confirm the predicting effects of resilience. A cross-sectional study in China showed that resilience played an important role among medical students and resilience mediated the effect of stress on life satisfaction, an indicator of psychological well-being [20]. Such findings suggested that students with high resilience were more likely to withstand stress and to achieve life satisfaction. There are few possible explanations for such positive findings. As a personality trait, resilience is envisioned as assets (such as intellectual functioning) that enable individuals to survive despite facing stressful situations and adversity [5]. Hence, undergraduate students might adjust to negative live events or other difficult circumstances (such as academic stress, relationship problems, and financial strain) and achieved PWB. As a process, resilience is perceived as an interactive dynamic process whereby resilience interacts with biological, psychological, social support, and social systems and contribute to positive health outcomes, including PWB [5].

### Strengths and limitations

This study has strengths concerning the use of SEM, which enabled simultaneous analyses of multiple independent and dependent variables while controlling for other independent variables. Therefore, the findings may reflect the true parameter estimates. Furthermore, we used two repeated cross-sectional samples and the parameter estimates were comparable across the two samples. Such findings provide strong evidence to support the hypothesized model of stress, resilience and PWB. However, our study contained limitations regarding the use of convenience sampling and samples were recruited from only one university. Therefore, generalizability of findings may be limited.

### Implications to clinical practice

Findings from this research provide stronger evidence to support the predicting effects of stress and reliance on both factors of PWB among university students. These findings have clinical implications and interventions aiming to reduce stress levels and to enhance resilience can be offered to the university students in the Philippines and other countries. Examples of stress management interventions include stress inoculation training, physical activity, progressive muscle relaxation, autogenic training, guided imagery relaxation, and mindfulness-based intervention. Additionally, resilience-based programs can be developed and offered to university students. A systematic review [30] showed that resilience can be cultivated through various avenues, encompassing interpersonal-based (such as Life Skill Training Program), family-focused (such as Adolescent Transition program and Iowa Strengthening Family Program), and community-based interventions that aim to enhance students’ engagement in broader social networks (such as community engagement program).

### Implications to future research

There is a need to further test the predicting effects of stress and resilience on PWB across difference assessment points using multi-center settings (such as multicultural settings). Intervention research such as randomized controlled trial (RCT) could be conducted to test the efficacy of stress management interventions and resilience-based programs to enhance PWB among university students in the Philippines and other countries. Additionally, future research should examine how biological (such as genetic-environment mechanisms), personal (such as personality traits and self-efficacy) and environmental factors (such as social support and relationships with others) play the role in the process of resilience-based programs.

## Conclusion

This study demonstrated that higher perceived stress and lower perceived control contributed to lower levels of PWB. However, resilience was associated with higher autonomy and growth and lower negative triad across the two repeated cross-sectional samples, approximately 1 year apart. This evidence supports the predicting effects of resilience. In this study, we utilized SEM to simultaneously analyze measurement and structural models. The factorial structures of all study variables were carefully explored prior to testing study hypotheses. Therefore, findings are perceived to accurately reflect true parameter estimates. Based on our findings, resilience-based interventions should be developed to help students manage their stress and enhance PWB. Future research (such as RCTs) should be carried out to test the effects of the interventions.

## Data Availability

The datasets generated and/or analysed during the current study are not publicly available but are available from the corresponding author on reasonable request.

## Abbreviations

PWB: Psychological Well-Being
RPF: Resource Protection Factors
SEM: Structural Equation Modeling
RMSEA: Root Mean Square Error of Approximation
PIS: Participant Information Sheet
PSS: Perceived Stress Scale
CD-RISC: Connor–Davidson Resilience Scale
PWBS: Psychological well-being Scale
CFI: Confirmatory Fit Index
IFT: Incremental Fit Index
TLI: Tucker-Luwis Index
